# Inhibition of the immunoproteasome ameliorates experimental autoimmune encephalomyelitis

**DOI:** 10.1002/emmm.201303543

**Published:** 2014-01-07

**Authors:** Michael Basler, Sarah Mundt, Tony Muchamuel, Carlo Moll, Jing Jiang, Marcus Groettrup, Christopher J Kirk

**Affiliations:** 1 Biotechnology Institute Thurgau (BITg) at the University of KonstanzKreuzlingen, Switzerland; 2 Division of Immunology, Department of Biology, University of KonstanzKonstanz, Germany; 3 Onyx PharmaceuticalsSouth San Francisco, CA, USA; 4 Institute of Pathology, Cantonal Hospital MuensterlingenMuensterlingen, Switzerland

**Keywords:** experimental autoimmune encephalomyelitis, immunoproteasome, multiple sclerosis, proteasome

## Abstract

Multiple sclerosis (MS) is a chronic demyelinating immune mediated disease of the central nervous system. The immunoproteasome is a distinct class of proteasomes found predominantly in monocytes and lymphocytes. Recently, we demonstrated a novel function of immunoproteasomes in cytokine production and T cell differentiation. In this study, we investigated the therapeutic efficacy of an inhibitor of the immunoproteasome (ONX 0914) in two different mouse models of MS. ONX 0914 attenuated disease progression after active and passive induction of experimental autoimmune encephalomyelitis (EAE), both in MOG_35–55_ and PLP_139–151_-induced EAE. Isolation of lymphocytes from the brain or spinal cord revealed a strong reduction of cytokine-producing CD4^+^ cells in ONX 0914 treated mice. Additionally, ONX 0914 treatment prevented disease exacerbation in a relapsing-remitting model. An analysis of draining lymph nodes after induction of EAE revealed that the differentiation to Th17 or Th1 cells was strongly impaired in ONX 0914 treated mice. These results implicate the immunoproteasome in the development of EAE and suggest that immunoproteasome inhibitors are promising drugs for the treatment of MS.

## Introduction

Multiple sclerosis (MS) is a complex chronic immune mediated disease of the central nervous system, affecting approximately 1 in 1000 individuals in Europe and the US (Rosati, 2001). Environmental and genetic factors determine the susceptibility to develop the disease that is characterized by acute relapses of neurological deficit and remissions, and by a progressive accumulation of neurological and cognitive disability over time. MS pathology was originally defined by the presence of focal white matter lesions, due to primary demyelination with partial preservation of axons and reactive astrocytic scar formation (Lassmann, 2005). Experimental autoimmune encephalomyelitis (EAE), a MS disease model in rodents, shares clinical and pathological features with MS (Whitehouse *et al*, 1969; Fuller *et al*, 2004; Sospedra & Martin, 2005; Croxford *et al*, 2011). In this model, sensitization with central nervous system (CNS) antigens breaks immunological tolerance of autoreactive T cells, enabling them to cross the blood brain barrier and induce brain inflammation.

The proteasome is a large intracellular multicatalytic protease found both in the cytoplasm and the nucleus controlling multiple cellular processes (Peters *et al*, 1994). The 20S proteasome is a barrel-shaped complex of four rings with seven subunits each. The outer two rings consist of α subunits, the inner two rings of β subunits forming the central proteolytic chamber (Huber *et al*, 2012). Three of the β subunits designated β1, β2, and β5 bear the active centers of the 20S proteasome. In cells stimulated with interferon-γ (IFN-γ) and tumor necrosis factor-α (TNF-α) or in cells of hematopoietic origin, the catalytic subunits LMP2 (β1i), MECL-1 (β2i), and LMP7 (β5i) replace the constitutive subunits β1, β2, and β5 during neosynthesis and build the so-called immunoproteasome. The immunological benefit of this process is attributed to minor structural changes within the proteasome (Huber *et al*, 2012) and an altered cleavage pattern, thus, the immunoproteasome optimizes quality and quantity of MHC class I presented peptides (Groettrup *et al*, 1995; Schwarz *et al*, 2000; Sijts *et al*, 2000; Toes *et al*, 2001). Thereby, the immunoproteasome alters class I ligand generation and, thus, shapes the naive CD8-T cell repertoire in the thymus and cytotoxic T cell responses in the periphery (Chen *et al*, 2001; Basler *et al*, 2004, 2006, 2011; Groettrup *et al*, 2010; Kincaid *et al*, 2012). Apart from its role in antigen presentation, we have recently shown that the immunoproteasome plays a crucial role in T cell expansion and autoimmune diseases (Muchamuel *et al*, 2009; Basler *et al*, 2010, 2013; Moebius *et al*, 2010). ONX 0914 (formerly named PR-957), an LMP7-selective epoxyketone inhibitor of the immunoproteasome, reduced cytokine production in activated monocytes or T cells and attenuated disease progression in mouse models of rheumatoid arthritis, diabetes, colitis, and systemic lupus erythematosus (Muchamuel *et al*, 2009; Basler *et al*, 2010; Ichikawa *et al*, 2012). Additionally, ONX 0914 blocked differentiation of naïve CD4^+^ T cells to IL-17 producing cells *in vitro* (Muchamuel *et al*, 2009).

These results prompted us to investigate the clinical effect of ONX 0914 in two different mouse models of MS. In MOG_35–55_-and PLP_139–151_-induced EAE, ONX 0914 attenuated disease progression in diseased animals following immunization with autoantigen or transfer of autoreactive T cells. Blockade of LMP7 prevented infiltration of immune cells into the brain and spinal cord and diminished initial Th1 and Th17 differentiation. Thus, our results suggest that the inhibition of LMP7 holds potential as a novel approach for the treatment of MS in humans.

## Results

### EAE induction in immunoproteasome-deficient mice

**Figure 1:**
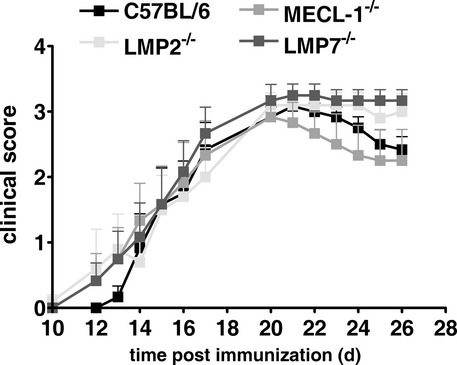
C57BL/6 wild type, LMP2^−/−^, MECL-1^−/−^, and LMP7^−/−^ mice were immunized with MOG_3__5__–__5__5_ peptide. Mice were monitored daily for clinical symptoms of EAE. Clinical score ( *y*-axis) is plotted versus time post immunization ( *x*-axis). Data points represent mean ± s.e.m. of six mice. The experiments were performed three times, yielding similar results.

In order to analyze whether mice deficient in immunoproteasome subunits are susceptible to experimental autoimmune encephalomyelitis, LMP2^−/−^, LMP7^−/−^, MECL-1^−/−^, and C57BL/6 control mice were immunized with MOG_35–55_ peptide. The clinical score of the mice was recorded for 26 days, but no significant difference in disease score could be observed (Fig 1). A similar finding was obtained by Frausto *et al* (2007) in LMP2-deficient mice, whereas Seifert *et al* (2010) reported an exacerbation of EAE symptoms in LMP7^−/−^ mice. However, the latter finding could not be confirmed by others (Nathan *et al*, 2013).

### An LMP7-selective inhibitor prevents symptoms of EAE

**Figure 2:**
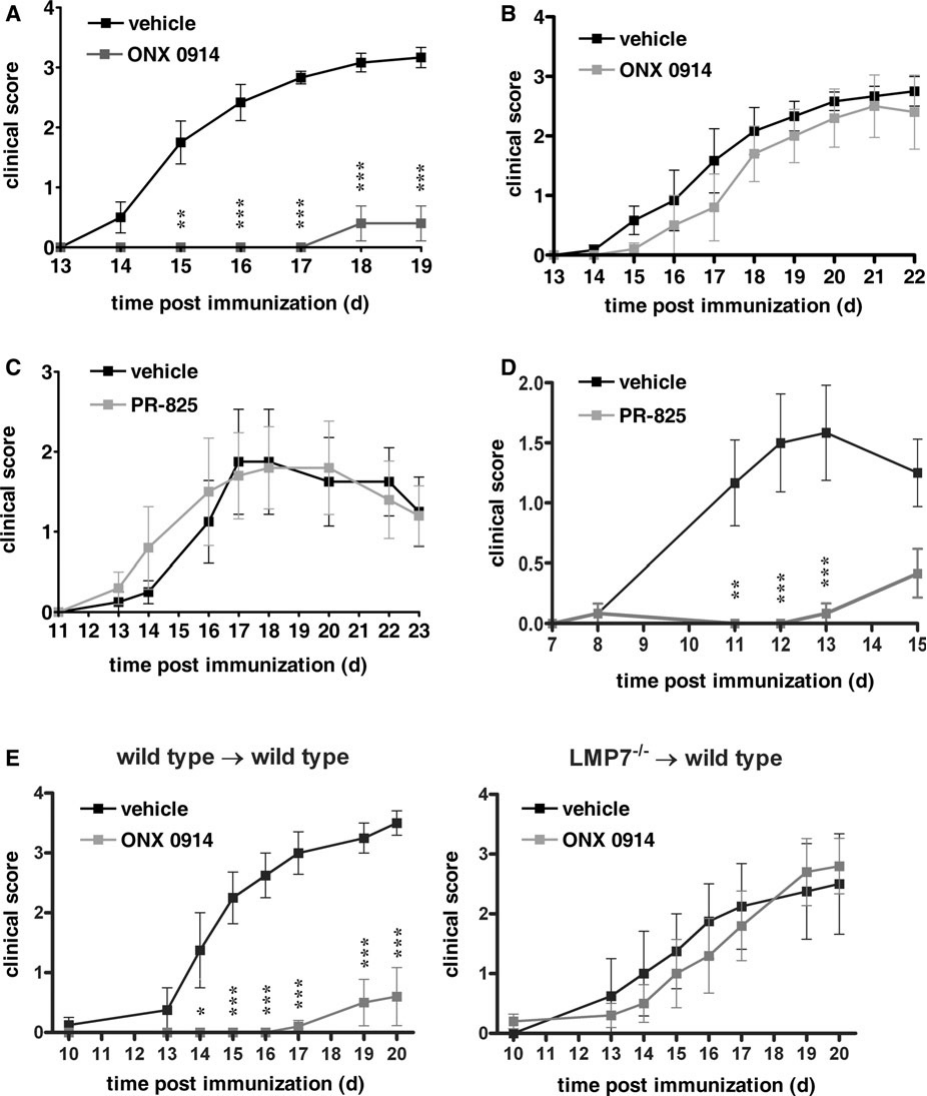
ONX 0914 prevents MOG35–55-induced EAE.Mice were immunized with MOG_35–55_ peptide and were monitored daily for clinical symptoms of EAE. Data are presented as mean clinical score s.e.m. of six mice. * *P *< 0.05; ** *P *< **0.01; *** *P *< **0.001. C57BL/6 mice were treated with 10 mg/kg ONX 0914 or vehicle three times a week beginning on the day of immunization. The experiments were performed five times, yielding similar results.LMP7^−/−^ mice were treated with 10 mg/kg ONX 0914 or vehicle three times a week beginning on the day of immunization. The experiments were performed twice, yielding similar results.C57BL/6 mice were treated with 2 mg/kg PR-825 or vehicle three times a week beginning on the day of immunization. The experiments were performed twice, yielding similar results.LMP7^−/−^ mice were treated with 1 mg/kg PR-825 or vehicle three times a week beginning on the day of immunization. The experiments were performed twice, yielding similar results.C57BL/6 wild type mice were irradiated and reconstituted with bone marrow derived from wild type (wild type → wild type) or LMP7^−/−^ (LMP7^−/−^ → wild type) mice. Mice were treated with 10 mg/kg ONX 0914 or vehicle, three times a week beginning on the day of immunization. The experiments were performed twice, yielding similar results.

ONX 0914 is a selective inhibitor of LMP7 with low nanomolar potency *in vitro* and at doses of 6–12 mg/kg in mice (Muchamuel *et al*, 2009; Basler *et al*, 2010; Huber *et al*, 2012). In order to investigate whether LMP7 inhibition affects clinical symptoms in MOG_35–55_-immunized mice, ONX 0914 was administered three times a week at an LMP7-selective concentration of 10 mg/kg (Muchamuel *et al*, 2009) beginning on the day of immunization. First clinical symptoms were observed on day 14 in vehicle treated mice, whereas ONX 0914 treatment resulted in a significantly later onset of the disease and lower disease severity (Fig 2A). Disease incidence in vehicle treated mice was 90%, compared to 23% in ONX 0914 treated mice (Table 1). This effect was due to specific inhibition of LMP7 as treatment of MOG_35–55_-immunized LMP7-deficient mice with ONX 0914 developed disease in a similar manner to either wild type or gene-deficient animals treated with vehicle (Fig 2B). When wild type mice immunized with MOG_35–55_ were treated with the β5c-selective inhibitor PR-825 (described in (Muchamuel *et al*, 2009)) no change in disease onset or severity was noted (Fig 2C). To address the question why an LMP7-selective inhibitor is so effective in attenuating symptoms of EAE (Fig 2A, Table 1), while LMP7^−/−^ are susceptible to the disease (Fig 1), EAE was induced in PR-825 treated LMP7-deficient mice. Interestingly, in contrast to wild type mice (Fig 2C) PR-825 treatment in LMP7^−/−^ mice resulted in a significantly later onset of the disease and lower disease severity (Fig 2D). Taken together, these data suggest that inhibition of LMP7 results in reduced clinical symptoms in MOG_35–55_-induced EAE.

**Table 1:** Clinical parameters of vehicle and ONX 0914 treated mice during MOG_3__5__–__5__5_-induced EAE^a^

Group	Day of disease onset (mean ± s.e.m.)^b^	Incidence (%)	Clinical score at the peak of disease (mean ± s.e.m.)
Vehicle	17.2 ± 0.7	27/30 (90)	2.6 ± 0.1
10 mg/kg ONX 0914	26.6 ± 1.1	6/26 (23.1)	1 ± 0.2

a Results are cumulative data from five different experiments.

b Not affected animals were arbitrary assigned to day 30 for disease onset (end of experiment).

To assess whether tissue cells or cells derived from the hematopoietic system are affected by ONX 0914 treatment (Fig 2A) we generated bone marrow chimeras. Wild type mice were irradiated and reconstituted with either wild type or LMP7-deficient bone marrow. Mice were immunized with MOG_35–55_ and ONX 0914 was administered three times a week at an LMP7-selective concentration of 10 mg/kg (Fig 2E). ONX 0914 treatment strongly reduced the clinical score in mice reconstituted with wild type bone marrow but not in wild type mice reconstituted with LMP7-deficient bone marrow. Hence, we conclude that the observed amelioration of EAE symptoms in ONX 0914 treated mice relies on the selective inhibition of LMP7 in cells derived from the hematopoietic system.

### LMP7 inhibition prevents brain and spinal cord inflammation

**Figure 3:**
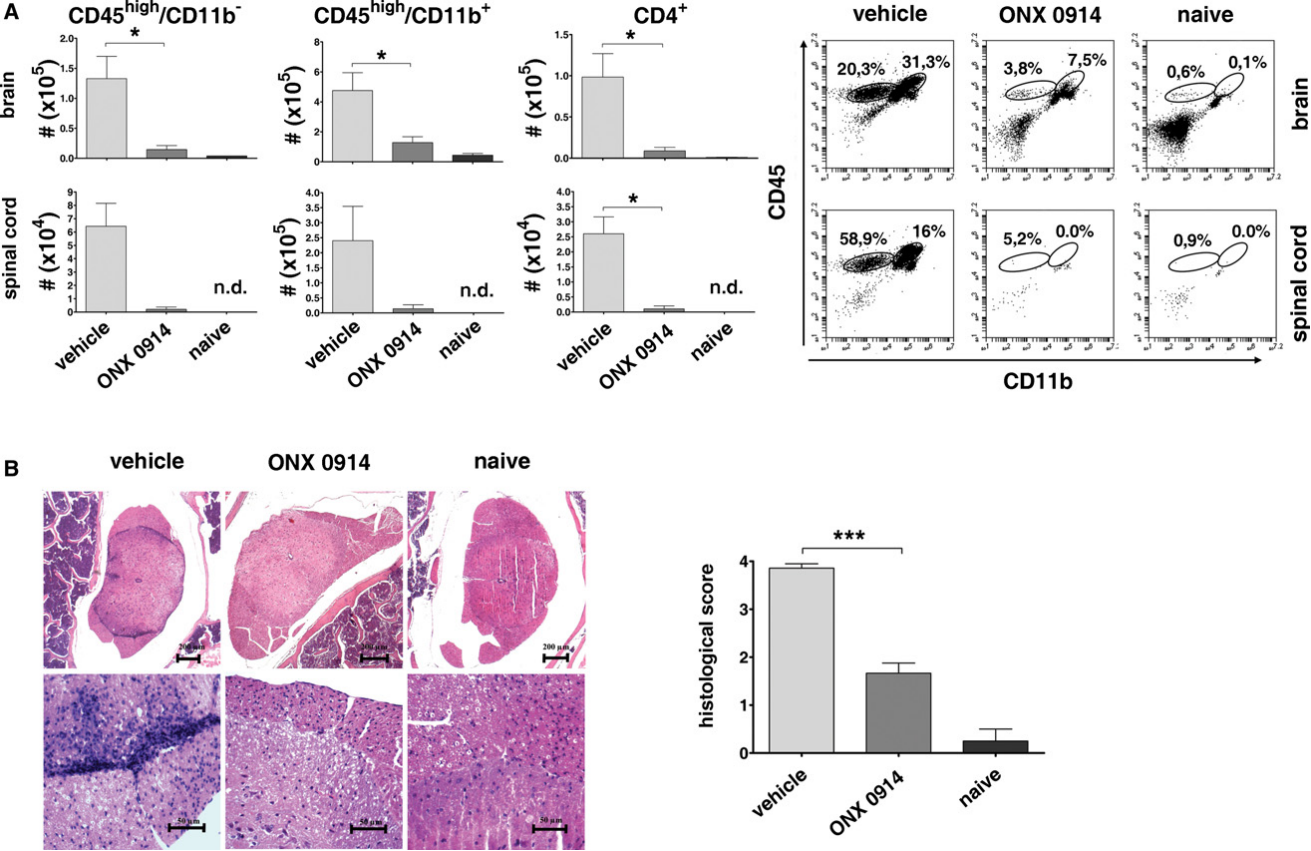
Reduced infiltration into the CNS in ONX 0914 treated mice.C57BL/6 mice were immunized with MOG_3__5__–__5__5_ peptide, treated three times a week with 10 mg/kg ONX 0914 or vehicle beginning on the day of immunization and analyzed on day 19 post immunization. The experiments were performed twice ( *n* = 6 per group and *n* = 2 naïve mice), yielding similar results. * *P* < 0.05; ** *P* < 0.01; *** *P* < 0.001. Flow cytometric analysis of lymphocytes and myeloid cells invading the brain (upper panels) or spinal cord (lower panels). Graphs show the mean absolute numbers ± s.e.m. of CNS invading CD4^+^ lymphocytes, CD45^high^CD11b^−^ lymphocytes, and CD45^high^CD11b^+^ myeloid cells. Representative flow cytometry profiles of CNS infiltrating cells are depicted on the right side. n.d.: not detected.Representative histological spinal cord sections (left side) of indicated mice (H&E, original magnification ×5 [upper panels] and ×40 [lower panels]). Semiquantitative histopathologic assessment (right side) of CNS infiltration. Data points represent mean ± s.e.m.

**Figure 4:**
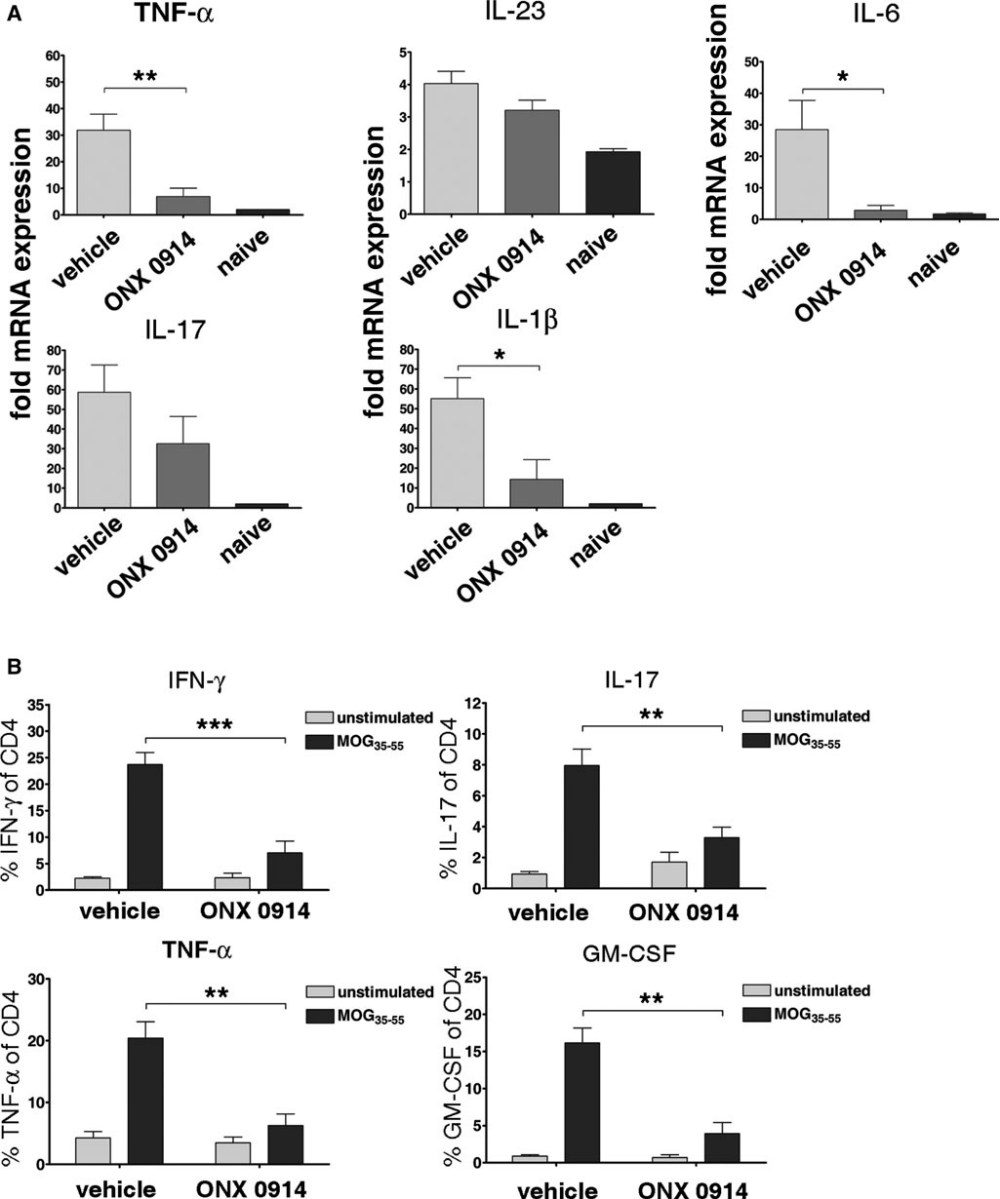
Reduced CNS inflammations in ONX 0914 treated mice.C57BL/6 mice were immunized with MOG_3__5__–__5__5_ peptide, treated three times a week with 10 mg/kg ONX 0914 or vehicle beginning on the day of immunization and analyzed on day 19 post immunization. The experiments were performed twice ( *n* = 6 per group and *n* = 2 naïve mice), yielding similar results. * *P* < 0.05; ** *P* < 0.01; *** *P* < 0.001. The TNF-α, IL-23, IL-17, IL-1β, and IL-6 mRNA content in spinal cords was analyzed by real-time RT–PCR. The values were normalized to the expression of hypoxanthineguanine phosphoribosyl transferase in the same organs. Shown are the mean fold expression ± s.e.m.B Brain infiltrating CD4^+^ lymphocytes were restimulated *in vitro* with MOG_35–55_ peptide for 6 h and analyzed by flow cytometry after staining for CD4 and intracellular IFN-γ, IL-17, TNF-α, or GM-CSF. Shown are the percentages of IFN-γ-, IL-17-, TNF-α-, or GM-CSF-positive cells of CD4^+^ T cells ( *y*-axis) as determined by flow cytometry. Unstimulated cells (no peptide) were used as a negative control.

Experimental autoimmune encephalomyelitis is characterized by the invasion of autoreactive T helper cells into the CNS, leading to inflammation and demyelination. Infiltration of immune cells into the brain and spinal cord on day 19 of MOG_35–55_-induced EAE was measured by flow cytometry. Inhibition of LMP7 reduced infiltration of CD4^+^ T helper cells, activated lymphocytes (CD45^high^CD11b^−^), and activated myeloid cells (CD45^high^CD11b^+^) (Fig 3A). The degree of inflammation in H&E stained cross-sections of the spinal cord was microscopically assessed and semiquantitatively graded from 0 to 4 blinded to sample identity (Fig 3B). In contrast to vehicle treated mice, ONX 0914 treated mice had only mild signs of inflammation. Several soluble mediators of inflammation, like proinflammatory cytokines, are elevated in EAE and play a crucial role in the course of the disease (Petermann & Korn, 2011). To investigate whether ONX 0914 treatment altered cytokine expression in mice after EAE induction, the mRNA levels for TNF-α, IL-1β, IL-6, IL-17, and IL-23 were determined by real-time RT–PCR in spinal cords on day 19 after immunization with MOG_35–55_ peptide (Fig 4A). Cytokines were upregulated in vehicle treated mice, whereas ONX 0914 treated mice showed significantly reduced TNF-α, IL-1β, and IL-6 mRNA production. To investigate whether autoreactive T helper cells of ONX 0914 treated mice were able to promote and sustain inflammation, we analyzed CD4^+^ T cells from the brains and spinal cords for their cytokine expression profile. While CD4^+^ cells derived from the brain of vehicle treated mice produced IFN-γ, TNF-α, IL-17, and GM-CSF upon *in vitro* restimulation with MOG_35–55_ (Fig 4B), the few CD4^+^ cells recovered from the brains of ONX 0914 treated mice were barely expressing these cytokines. Although some CD4^+^ T cells were able to invade the brain of ONX 0914 treated mice, these data suggest that LMP7 inhibition dampens the ability of these cells to produce cytokines.

**Figure 5:**
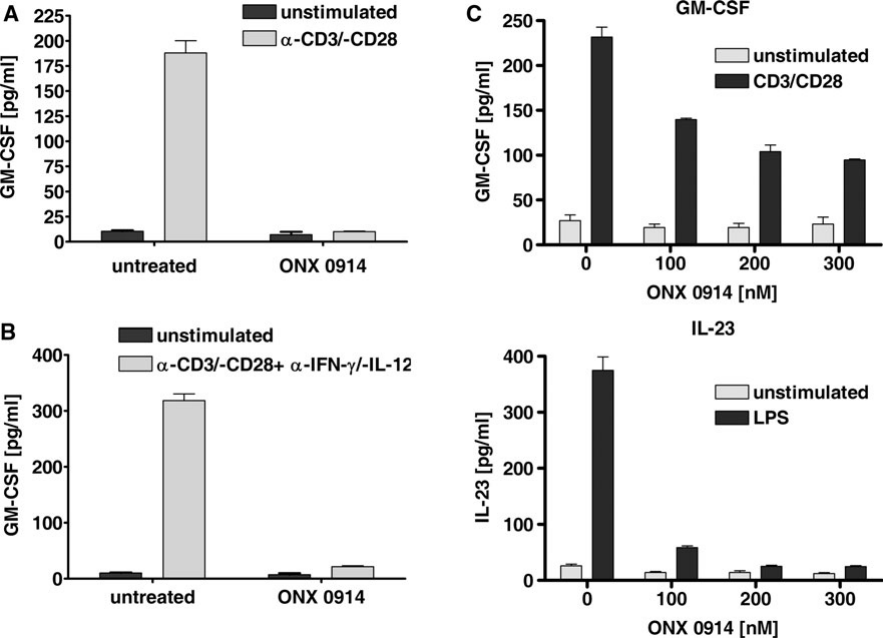
ONX 0914 blocks GM-CSF production of T cells.Cytokine concentrations measured by ELISA are presented as the mean s.e.m. from triplicate wells. Data represent one out of three experiments. GM-CSF production of ONX 0914 (300 nM) treated splenocytes stimulated with plate bound anti-CD3/anti-CD28 Abs for 96 h as analyzed by ELISA.GM-CSF production of ONX 0914 (300 nM) treated splenocytes stimulated with plate bound anti-CD3/anti-CD28 Abs in the presence of neutralizing Abs to IFN-c and IL-12 for 96 h as analyzed by ELISA.Assessment of GM-CSF and IL-23 production of human PBMCs. PBMCs were incubated with indicated concentrations of ONX 0914 for 2 h and stimulated with plate bound anti-CD3/anti-CD28 Abs (GM-CSF) or LPS (IL-23) for 48 h.

Recently, we have demonstrated that LMP7 regulates secretion of IL-23 and IL-6 by activated monocytes and IFN-γ and IL-2 by T cells (Muchamuel *et al*, 2009). GM-CSF has been previously reported to play a pivotal role in the initiation of autoimmune neuroinflammation (Codarri *et al*, 2011; El-Behi *et al*, 2011). To investigate whether LMP7 inhibition is able to influence the secretion of this crucial cytokine in neuroinflammation, splenocytes were stimulated with plate bound CD3/CD28 antibodies in the presence or absence of ONX 0914 and GM-CSF secretion was measured 24 h later in the supernatant by ELISA (Fig 5A). LMP7-selective inhibition with ONX 0914 reduced the secretion of GM-CSF to background levels. A similar result was found when T cells were stimulated with plate bound CD3/CD28 antibodies under GM-CSF polarizing conditions (i.e. in the presence of neutralizing Abs to IFN-γ and IL-12) for 96 h as previously reported (Fig 5B) (Codarri *et al*, 2011). ONX 0914 treatment at 300 nM reduced cytokine secretion to background levels. To determine whether ONX 0914 can inhibit GM-CSF and IL-23 secretion of human cells, human PBMCs were incubated with varying concentrations of ONX 0914. Twenty-four hours post stimulation with plate bound CD3/CD28 antibodies, GM-CSF secretion into the supernatant was analyzed by ELISA whereas IL-23 production was assessed after LPS stimulation of PBMCs. ONX 0914 reduced IL-23 secretion to background levels at 100 nM (Fig 5C bottom panel) in accordance with our previous report (Muchamuel *et al*, 2009), while it diminished GM-CSF production by human PBMCs in a dose-dependent manner to approximately 50% at 300 nM.

### Therapeutic treatment with ONX 0914 inhibits progression of MOG_3__5__–__5__5_-induced EAE

**Figure 6:**
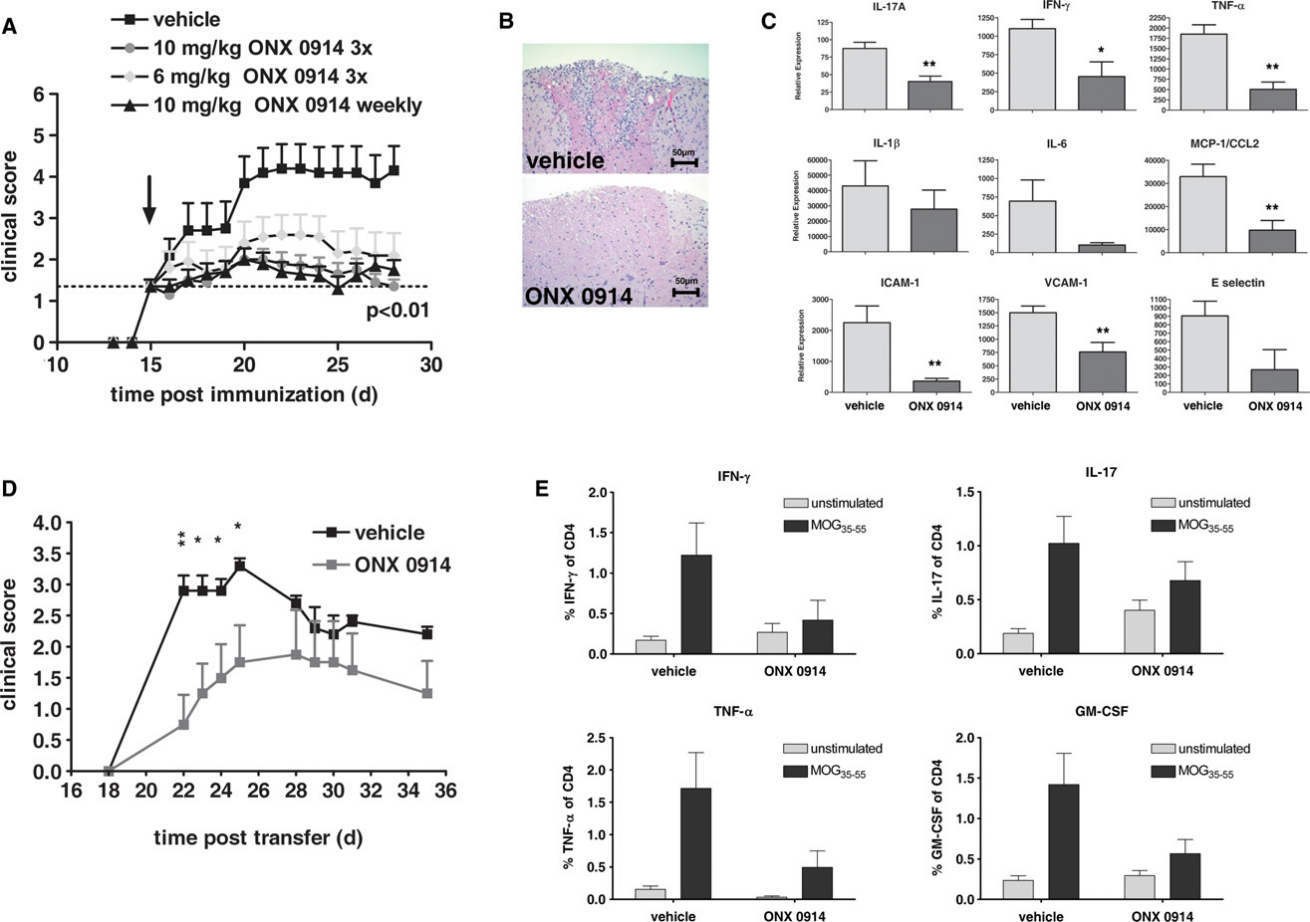
ONX 0914 inhibits progression of MOG35–55-induced EAE. C57BL/6 were immunized with MOG_3__5__–__5__5_ peptide and were daily scored for clinical symptoms. On the day of disease onset (d 15) mice were treated three times a week with intravenous administration of 6 mg/kg ONX 0914, 10 mg/kg ONX 0914, or vehicle or once a week with 10 mg/kg ONX 0914. Data, presented as the mean clinical score ± s.e.m. ( *n* = 10 per group), are from one experiment of three performed with similar results. The arrow indicates the time point when treatment was initiated. * *P* < 0.05; ** *PA * < 0.01; *** *P* < 0.001.Histopathological analysis of spinal cords from MOG_3__5__–__5__5_-immunized mice at day 25 after immunization. Data are a representative of 2 separate experiments.MOG_3__5__–__5__5_-immunized mice received either vehicle or 10 mg/kg ONX 0914 three times per week starting on day 14 after immunization. Individual spinal cords were harvested on day 25 from a cohort of animals in each group and analyzed by quantitative RT–PCR (β-actin normalized) for expression of the indicated genes. Data presented are the mean normalized value ± s.e.m. ( *n* = 5 per group) and *P* values were derived from an unpaired *t*-test.*In vitro* restimulated MOG_3__5__–__5__5_ reactive T cells were adoptively transferred into C57BL/6 mice. Mice were treated three times a week with 6 mg/kg ONX 0914 or vehicle for 14 days and were daily monitored for clinical symptoms. Data are presented as mean clinical score ± s.e.m. ( *n* = 5 per group).C57BL/6 were immunized with MOG_3__5__–__5__5_ peptide and treated with 10 mg/kg ONX 0914 or vehicle, three times a week beginning on the day of immunization. On day 9 post immunization draining lymph node cells were restimulated *in vitro* with MOG_3__5__–__5__5_ peptide for 6 h and analyzed by flow cytometry after staining for CD4 and intracellular IFN-γ, IL-17, TNF-α, or GM-CSF. Shown are the mean percentages ± s.e.m. ( *n* = 6 per group) of IFN-γ-, IL-17-, TNF-α-, or GM-CSF-positive cells of CD4^+^ T cells ( *y*-axis) as determined by flow cytometry. Unstimulated cells (no peptide) were used as a negative control.

In order to investigate whether ONX 0914 is able to ameliorate active EAE, mice were immunized with MOG_35–55_ peptide. When the first clinical symptoms were visible (day 15), mice were randomized and treated with vehicle or ONX 0914 either once or three times per week. Treatment with ONX 0914 prevented progression to severe disease (Fig 6A) and resulted in reduced cellular infiltration into the spinal cord (Fig 6B). In addition, expression of several inflammatory cytokines and adhesion molecules were reduced in the ONX 0914 treated animals as compared to vehicle control animals (Fig 6C). Next, splenocytes from diseased EAE mice were restimulated *in vitro* with MOG_35–55_ peptide and adoptively transferred into C57BL/6 mice. Treatment of these animals at the time of transfer with ONX 0914 significantly reduced disease onset and severity compared to vehicle treated mice (Fig 6D). These results indicate that LMP7 inhibition suppresses fully primed autoreactive T cells and prevents autoimmunity.

### ONX 0914 blocks differentiation to autoreactive T cells

Recently, we have shown that ONX 0914 blocked Th17 cell differentiation of T cells cultured in the presence of polarizing cytokines (Muchamuel *et al*, 2009). Th17 cells can be isolated from the inflamed CNS of EAE mice and their signature cytokine, IL-17, can be measured in MS lesions (Lock *et al*, 2002). Barely any cytokine producing CD4^+^ cells were found in the brain of ONX 0914 treated MOG_35–55_-immunized mice (Fig 4B). To investigate whether the initial differentiation to Th1 or Th17 cells is altered in MOG_35–55_-immunized mice treated with ONX 0914, cells from draining lymph nodes were restimulated *in vitro* with MOG_35–55_ peptide and analyzed for TNF-α, GM-CSF, IFN-γ, and IL-17 expression (Fig 6E). In contrast to vehicle treated animals, almost no MOG_35–55_-specific IFN-γ or IL-17 producing CD4^+^ cells could be recovered from mice treated with ONX 0914. In addition, antigen-specific expression of TNF-α and GM-CSF were strongly reduced in CD4^+^ T cells of ONX 0914 treated mice. Thus, it appears that the differentiation to Th1 and Th17 cells *in vivo* is blocked in mice treated with an LMP7-selective inhibitor.

### ONX 0914 reduces clinical symptoms in the PLP_1__3__9__–__1__5__1_-induced relapsing-remitting EAE model in SJL/J Mice

**Figure 7:**
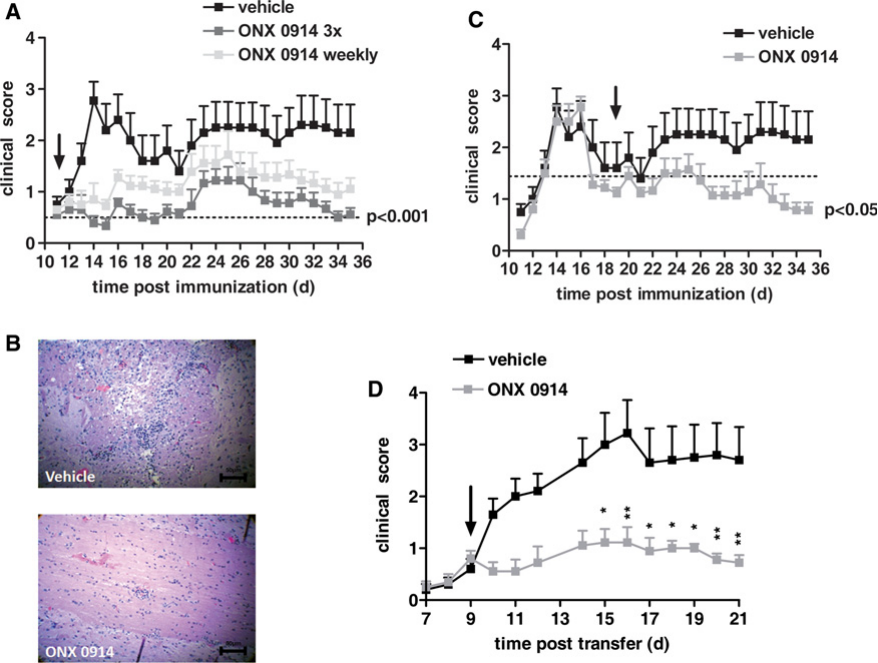
ONX 0914 ameliorates PLP139–151-induced EAE. SJL/J mice were immunized with PLP_139–151_ and were monitored daily for clinical symptoms of EAE. From day 11 on (indicated by an arrow), mice were treated with 10 mg/kg ONX 0914 three times a week (indicated 3×), 10 mg/kg ONX 0914 once a week (indicated weekly), or vehicle. Data, presented as the mean clinical score s.e.m. ( *n* = 10 per group), are from one experiment of three performed with similar results. *** *P *< **0.001 by two-way ANOVA followed by Bonferroni post-hoc comparison at the end of study.Representative histological spinal cord sections of indicated mice. From day 11 on, mice were treated with10 mg/kg ONX 0914 three times a week. Mice were analyzed on day 14 post immunization.SJL/J mice were immunized with PLP_139–151_ and were monitored daily for clinical symptoms of EAE. From day 19 on (indicated by an arrow), mice were treated with 10 mg/kg ONX 0914 three times a week (indicated 3×), 10 mg/kg ONX 0914 once a week (indicated weekly), or vehicle. Data, presented as the mean clinical score s.e.m. ( *n* = 10 per group), are from one experiment of three performed with similar results. *** *P *< **0.001 by two-way ANOVA followed by Bonferroni post-hoc comparison at the end of study.In vitro restimulated PLP_139–151_–reactive T cells were adoptively transferred into SJL/J mice. Mice were treated three times a week with 10 mg/kg ONX 0914 or vehicle beginning on day 9 and were monitored daily for clinical symptoms. Data are presented as mean clinical score s.e.m. ( *n* = 9–10 per group). * *P *< **0.05; ** *P *< **0.01; *** *P *< **0.001.

Immunization of SJL/J mice with PLP_139–151_ induces a relapsing-remitting EAE that resembles the relapsing-remitting form of MS in humans (McRae *et al*, 1992). In this model, mice fully or almost fully recover from the first wave of paralysis after immunization and after a disease-free period of 1–2 weeks, 50–100% of the mice develop a second wave of paralysis. When clinical symptoms were visible, animals were treated once or three times a week with ONX 0914 or vehicle. Treatment with ONX 0914 almost completely blocked the first wave of paralysis in these mice (Fig 7A). Interestingly, a relapse was noted in inhibitor treated mice but was significantly lower compared to vehicle treated animals. In accordance with the clinical symptoms, reduced cellular infiltration into the spinal cord could be observed by microscopic assessment of cross-sections of the spinal cord of ONX 0914 treated mice (Fig 7B). To investigate whether a relapse could be prevented in the PLP-induced mouse model of EAE, PLP_139–151_-immunized SJL/J mice were randomized on day 19 after the first wave of paralysis and were then treated with either ONX 0914 or vehicle (Fig 7C). In contrast to the vehicle treated mice, no relapse was observed in the ONX 0914 treated group. Hence, both disease progression and relapse can be prevented by LMP7 inhibition in this mouse model of MS.

We have demonstrated that administration of ONX 0914 prevented EAE symptoms after transfer of MOG_35–55_-specific autoreactive T cells (Fig 6D). To confirm this result in the PLP model of EAE, draining lymph nodes from PLP_139–151_-immunized mice were removed on day 10 post immunization. Cells were restimulated *in vitro* in the presence of PLP_139–151_ peptide and polarized to a Th17 phenotype in the presence of IL-23. The Th17 status was verified by both cytokine production and intracellular staining of RORγT and IL-17 (supplementary Fig S1). *In vitro* restimulated cells were then adoptively transferred into SJL/J mice and were randomized to treatment with either vehicle or ONX 0914 following the onset of clinical symptoms. Severe EAE developed in vehicle treated animals but not in the ONX 0914 treated mice (Fig 7D). Since treatment of the PLP_139–151_ T cells with ONX 0914 prior to restimulation prevented the production of IL-17 or IL-22 (data not shown), even in the presence of IL-23, it is likely that the *in vivo* effect is due to a blockade in Th17 function in treated animals.

## Discussion

Proteasome inhibitors represent novel drugs in anti-cancer therapy as they can induce apoptosis preferentially in transformed cells (Delic *et al*, 1998; Orlowski *et al*, 1998), but their therapeutic potential may also extend to autoimmune diseases due to their effects on cytokine production and proliferating lymphocytes (Bennett & Kirk, 2008). Bortezomib, which targets both the constitutive proteasome (CP) and the immunoproteasome (IP), is the first clinically approved proteasome inhibitor and is used in the treatment of multiple myeloma and relapsed mantle cell lymphoma (Adams *et al*, 1999). Apart from its direct anti-tumor activity against malignant B-cells, bortezomib eliminates both short-and long-lived plasma cells probably by activation of the terminal unfolded protein response (Neubert *et al*, 2008). Treatment with bortezomib depleted plasma cells producing antibodies to double-stranded DNA, eliminated autoantibody production, ameliorated glomerulonephritis, and prolonged survival the NZB/W F1 and MRL/lpr mouse models of lupus nephritis. To overcome resistance to bortezomib and to develop inhibitors with improved pharmacological and toxicological profiles, the development of proteasome inhibitors with enhanced selectivity is warranted. Therefore, novel proteasome inhibitors preferentially targeting subunits of the immunoproteasome were developed (Ho *et al*, 2007; Van Swieten *et al*, 2007; Kuhn *et al*, 2009; Muchamuel *et al*, 2009; Singh *et al*, 2011). The selectivity of proteasome inhibitors is determined by the geometry and amino acid side chain properties of the substrate-binding pocket, which differs between the three catalytic beta subunits (Groll *et al*, 2006). Recently, we have solved the crystal structures of the constitutive proteasome and immunoproteasome of the mouse at 2.9 Å resolution (Huber *et al*, 2012). These data will promote the structure-guided design of inhibitory lead structures selective for immunoproteasomes.

In this study, we investigated the efficacy of the LMP7-selective epoxyketone proteasome inhibitor ONX 0914 (formerly named PR-957) in two different mouse models for EAE. In MOG_35–55_-induced EAE, ONX 0914 reduced clinical symptoms of EAE and prevented brain and spinal cord inflammation (Figs 4). Interestingly, we found that mice genetically deficient in any of the immunoproteasome active site subunits developed EAE following MOG_35–55_ immunization that was indistinguishable from wild type C57BL/6 mice (Fig 1). Similar results using LMP2^−/−^ mice have been reported by Frausto *et al* (2007) while Seifert *et al* have reported that MOG_35–55_ immunization of LMP7^−/−^ mice results in more severe inflammation compared to wild type mice (Seifert *et al*, 2010), which could not be confirmed by others (Nathan *et al*, 2013). Although a precise reason for these disparate results is not known, it appears that the clinical severity reported by Seifert *et al* (approximately 0.75) was much lower than we report here. Nonetheless, when wild type animals are immunized with MOG_35–55_ and treated with an LMP7-selective inhibitor, disease onset was delayed and overall severity was reduced. The β5c-selective inhibitor PR-825 failed to prevent disease induction in C57BL/6 wild type mice (Fig 2C). In contrast, LMP7-deficient mice treated with the same inhibitor were almost completely protected from MOG_35–55_-induced EAE (Fig 2D). Hence, we can experimentally explain the discrepancy in phenotype between drug treated mice (Fig 2A) and immunoproteasome knockout mice (Fig 1). Namely, the chymotrypsin-like activity of the proteasome is responsible for the observed beneficial effects of the LMP7-selective inhibitor ONX 0914 on EAE. It seems that the cells responsible for the induction of EAE express high levels of immunoproteasomes. Therefore, the chymotrypsin-like activity in wild type cells (which contain LMP7) can only be blocked with ONX 0914, whereas in LMP7-deficient mice which incorporate β5c instead of LMP7 into immunoproteasomes, this activity can be inhibited by the β5c-specific inhibitor PR-825. Consistent with this is the finding that the β5c-specific inhibitor PR-825 suppresses production of IFN-γ or IL-17 in stimulated T cells from LMP7^−/−^ but not wild type mice, whereas ONX 0914 inhibits cytokine production only in T cells from wild type animals (Basler *et al*, 2010). The finding that the chymotrypsin-like activity of the proteasome is responsible for EAE prevention explains why broad-spectrum proteasome inhibitors are effective in EAE prevention (Vanderlugt *et al*, 2000; Hosseini *et al*, 2001; Fissolo *et al*, 2008). Nevertheless, these inhibitors are probably of no clinical relevance for the treatment of autoimmune diseases due to their harmful side effects. Bortezomib, a proteasome inhibitor for relapsed and/or refractory myeloma and mantle cell lymphoma, is used at the maximal tolerated dose (Aghajanian *et al*, 2002; Orlowski *et al*, 2002). In contrast, the dose of ONX 0914 used in this manuscript to prevent EAE (10 mg/kg) is far below the maximal tolerated dose of 30 mg/kg (Muchamuel *et al*, 2009). Thereby, ONX 0914 is selectively targeting the chymotrypsin-like activity in cells originating from the hematopoietic system (like T cells, dendritic cells, or myeloid cells) which mainly contain immunoproteasomes and these cells are responsible in the induction of EAE. Indeed, experiments with bone marrow chimeras demonstrated that the effect of LMP7 inhibition in cells derived from the hematopoietic system is responsible for the amelioration of EAE symptoms in ONX 0914 treated mice (Fig 2E). Hence, ONX 0914 is selectively targeting the chymotrypsin-like activity at the site of inflammation and, therefore, the chymotrypsin-like activity required for housekeeping functions of the proteasome at other sites in the body are not affected. This also explains the lacking side effects of ONX 0914 in mice compared to broad-spectrum proteasome inhibitors. We even demonstrated that a β5c selective inhibitor (PR-825) of the constitutive proteasome had no effect on EAE (Fig 2C). Therefore, our data demonstrate that selective inhibition of LMP7 results in a dramatically improved safety margin over non-selective inhibitors. This study describes for the first time LMP7 as a prime pharmacological target in the prevention of EAE. In contrast to EAE, immunoproteasome-deficient mice are protected from dextran sulfate sodium (DSS)-induced colitis (Fitzpatrick *et al*, 2006; Basler *et al*, 2010; Schmidt *et al*, 2010). Hence, it appears that LMP7 plays a different role in this disease model.

EAE is characterized by the infiltration of lymphocytes and myeloid cells into the CNS. Selective inhibition of LMP7 reduced migration of CD4^+^ T helper cells, activated lymphocytes (CD45^high^CD11b^−^), and activated myeloid cells (CD45^high^CD11b^+^) into the brain and spinal cord of MOG_35–55_-immunized mice (Fig 3A). Inflammation was reduced (Fig 3B) and the production of numerous cytokines was strongly impaired in ONX 0914 treated mice (Fig 4A). In addition, CD4^+^ cells isolated on day 19 post MOG_35–55_ immunization from the brain of ONX 0914 treated mice barely produced IFN-γ, TNF-α, IL-17, or GM-CSF upon *in vitro* restimulation with MOG_35–55_ peptide (Fig 4B).

Recent work has demonstrated roles for GM-CSF and IL-23 in MS. T cell-derived GM-CSF sustains neuroinflammation via myeloid cells that infiltrated the CNS (Codarri *et al*, 2011; El-Behi *et al*, 2011) and IL-23 is necessary for the induction of EAE in mice via its role in the maintenance of Th17 cells (Becher & Segal, 2011). GM-CSF is thought to stimulate the production of IL-23 by dendritic cells during autoimmune responses in a CCR4-dependent manner (Poppensieker *et al*, 2012). ONX 0914 reduced GM-CSF production of activated mouse T cells under GM-CSF polarizing and non-polarizing conditions and in human PBMC stimulated through the T cell receptor (Fig 5). Additionally, ONX 0914 blocked IL-23 (Muchamuel *et al*, 2009; and Fig 5C) production of human PBMCs and the differentiation of CD4^+^ cells to IFN-γ producing Th1 and IL-17 producing Th17 cells (Fig 6E). We have recently shown that Th17 differentiation under lineage promoting polarizing conditions *in vitro* is diminished in ONX 0914 treated CD4^+^ T cells (Muchamuel *et al*, 2009). Taken together, this suggests that systemic immunoproteasome inhibition has direct and indirect effects on autoreactive inflammatory T cells.

Th17 cells are involved in the onset and maintenance of EAE (Steinman, 2007). IL-17^+^ T cells have been found in lesions in brain tissues from patients with MS, indicating that Th17 cells also play a crucial role in the human demyelinating disease (Tzartos *et al*, 2008). It has been demonstrated that C-C chemokine receptor 6 (CCR6)-regulated entry of Th17 cells into the CNS through the choroid plexus is required for the initiation of EAE which may explain why ONX 0914 mediated blockage of Th17 differentiation is extremely efficient in the suppression of EAE (Fig 6E; and Muchamuel *et al*, 2009). Additionally, we found that silencing LMP7 inhibited Th1 differentiation and promoted regulatory T cell (T_reg_) development, whereas Th2 differentiation was not affected (Kalim *et al*, 2012). However, we did not detect a difference in the number of T_regs_ in the brains of ONX 0914 treated mice 19 days after MOG_35–55_ immunization (data not shown).

LMP7 inhibition was not only effective in protecting from EAE development (Figs 2A, 3, 4, 6E), but also prevented progression in diseased animals (Figs 6A–C, 7A–C). These results indicate that LMP7 blockade suppresses fully active and differentiated autoreactive cells. The clinical benefit can not be explained simply by the elimination of immune cells, because ONX 0914 treatment did not alter CD4^+^, CD8^+^, CD19^+^, and CD11c^+^ counts in the spleen and lymph nodes on day 19 post MOG_35–55_ immunization (data not shown). Since LMP7 inhibition blocks cytokine production in multiple immune effector cells, it is possible that ONX 0914 treatment prevents differentiation to and cytokine production by autoreactive T cells. Given that LMP7, but not β5 is required for cytokine release (Muchamuel *et al*, 2009; Basler *et al*, 2010, 2011; Hensley *et al*, 2010; Rockwell *et al*, 2012), we propose that the immunoproteasome selectively process a yet to be determined factor that is required for regulating cytokine production.

Consecutive episodes of remission and relapse are hallmarks of MS in the majority of patients. PLP_139–151_-induced relapsing-remitting EAE in SJL/J mice is an ideal model to study the relapse of the demyelinating disease. When ONX 0914 was administered on the day when the first clinical symptoms appeared, the inhibitor could prevent the initiation of the disease (Fig 7A). Additionally, LMP7 inhibition ameliorated a relapse when treatment started in the recovery phase after the first wave of symptoms (Fig 7C). Hence, LMP7 inhibition may qualify as a new treatment option to prevent the progression of autoimmune diseases like MS.

## Materials and Methods

### Mice, viruses, cell lines, and media

Female C57BL/6 mice (H-2^b^) were originally purchased from Charles River (Sulzfeld, Germany). Female SJL/J mice were obtained from Jackson Laboratories (Bar Harbor, ME, USA). MECL-1 (Basler *et al*, 2006), LMP2 (Van Kaer *et al*, 1994), and LMP7 (Fehling *et al*, 1994) gene-targeted mice were provided by John Monaco (University of Cincinnati, Cincinnati, OH). Mice were kept in a specific pathogen-free facility and used at 6-10 weeks of age. Animal experiments were approved by the review board of Regierungspräsidium Freiburg or were performed under protocols approved by an institutional animal care and use committee. All media were purchased from Invitrogen-Life Technologies (Karlsruhe, Germany) and contained GlutaMAX, 10% FCS, and 100 U/ml penicillin/streptomycin.

### Synthetic peptides

The synthetic peptide MOG_35 *–*55_ (MEVGWYRSPFSRVVHLYRNGK) was obtained from GenScript (Piscataway, NY, USA) or Bio-synthesis Inc. (Lewisville, TX, USA). PLP_139 *–*151_ (HSLGKWLGHPDKF) was purchased from Bio-synthesis Inc.

### Proteasome inhibitor

The β5c-(PR-825) and β5i-(ONX 0914; formerly called PR-957) (Onyx pharmaceuticals, South San Francisco, CA, USA) selective inhibitors were dissolved at a concentration of 10 mM in DMSO and stored at −20°C.

### Proteasome inhibition in mice

ONX 0914 was formulated in an aqueous solution of 10% (w/v) sulfobutylether-β-cyclodextrin and 10 mM sodium citrate (pH 6) and administered to mice as an s.c. or i.v. bolus dose of 6 or 10 mg/kg (in a volume of 100 μl). PR-825 was dissolved in 2% ethanol in saline and administered as an s.c. bolus dose of 2 mg/kg (in a volume of 100 μl) in C57BL/6 mice or 1 mg/kg in LMP7^−/−^ mice.

### Induction of EAE

C57BL/6 mice were immunized subcutaneously in the lateral abdomen with 300 μg MOG_35–55_ peptide in CFA and 200 ng pertussis toxin in PBS was administered on day 0 (i.p.) and day 2 (i.v.). Clinical disease was scored as follows: 0, no detectable signs of EAE; 0.5, distal limp tail; 1, complete limp tail; 1.5, limp tail and hind limb weakness; 2, unilateral partial hind limb paralysis; 2.5, bilateral partial hind limb paralysis; 3, complete bilateral hind limb paralysis; 3.5, complete hind limb paralysis and unilateral forelimb paralysis; 4, total paralysis of fore and hind limbs (score >4, to be killed); 5, death. For Fig 6A and B the following disease score was used: 0, no detectable signs of EAE; 1, tail weakness or tail paralysis or hind limb weakness but not both; 2, tail paralysis and hind leg weakness; 3, single hind leg paralysis. Noticeable gait disturbance, possible atonic bladder; 4, complete hind leg paralysis; 5, Complete hind leg and with partial or complete front leg paralysis; 6, moribund. Each time point shown is the average disease score of each group ± s.e.m.

For induction of PLP-induced EAE, female SJL/J (8–12 weeks) mice were immunized subcutaneously with 200 μg of PLP_139–151_ peptide dissolved in PBS and emulsified with an equivalent volume of CFA containing 2.5 mg/ml of *Mycobacterium tuberculosis* (Chondrex) under the skin at the nape of the neck and the right and left flank on day 0. Pertussis toxin (200 ng per mouse; List Biological Laboratories Inc., Campbell, CA, USA) was injected i.v. on the same day as the immunization.

### Generation of bone marrow chimeras

Age-and sex-matched C57BL/6 recipient mice were lethally irradiated with 9.8 Gy and received 3 × 10^6^ bone marrow cells from age-and sex-matched LMP7^−/−^ or C57BL/6 donor mice on the same day by i.v. injection. Mice were rested 8 weeks before use in experiments.

### Adoptive transfer

MOG_35–55_-immunized mice were sacrificed on day 11 post EAE induction. Spleen and lymph node cells were restimulated *in vitro* in the presence of 15 μg/ml MOG_35–55_ and 2.5 ng/ml IL-12 for 4 days. 1–3 × 10^7^ cells were injected (i.p.) into recipient mice and 200 ng pertussis toxin in PBS was administered (i.p.) on day 0 and day 2.

### Transfer in SJL/J mice

Donor SJL/J mice were immunized with PLP and CFA as described above and 8–10 days later, draining lymph nodes were removed. PLP-specific cells were cultured in RPMI 1640 supplemented with 10% FCS, 2 mM l-glutamine, 1% penicillin/streptomycin, 20 μg/ml of PLP, and 5 ng/ml of rIL-23 (R&D) for 4 days. Cells were washed and adjusted to the required concentration in PBS so that each naïve SJL/J recipient received 5 × 10^6^ cells i.v. in 0.1 ml. Recipient mice were monitored twice daily for clinical signs of disease

### Quantitative real-time RT–PCR

Real-time RT–PCR was used to quantify cytokine expression levels in mouse spinal cords. For Fig 4A, samples were disrupted in TRIzol® (Invitrogen) using a tissue homogenizer. After centrifugation chloroform was added. Total RNA was extracted from the aqueous phase using the Rneasy Plus Mini Kit including Optional On-Column Dnase Digestion with the RNase-Free Dnase Set (Qiagen, Valencia, CA, USA). One micro gram of total RNA was reverse transcribed using oligonucleotide (dT) primers and the reverse transcription system (Promega, Madison, WI, USA). Quantitative real-time PCR was performed with the LightCycler instrument, using the Light-Cycler Fast Start DNA Master SYBR Green I reaction mix (both from Roche Applied Science, Mannheim, Germany). Primers were previously described (Basler *et al*, 2010). Mouse hypoxanthineguanine phosphoribosyl transferase was used as a reference gene.

For Fig 6C, RNA isolation and polymerase chain reaction (PCR) analysis was performed by Gene Screen Technologies (Piscataway, NJ, USA). Total RNA was extracted by RNAeasy extraction kit (Qiagen). After treatment with DNase I (Qiagen), complementary DNA (cDNA) was synthesized using random primers and Superscript II as per the manufacturer's protocol (Invitrogen). Gene expression was measured by TaqMan realtime RT-PCR using target gene probes and primers per the manufacturer's protocol (Applied Biosystems, Grand Island, NY, USA). The experiments were performed on an ABI PRISM 7900 sequence detection system under the following conditions: 1 cycle of 50°C (2 min) followed by 95°C (10 min), 40 cycles of 95°C (15 s) followed by 60°C (1 min). All reactions were performed in triplicate and the experiments were repeated three times. Cytokine mRNA levels were normalized to β-actin.

**The paper explained**

**Problem**

Multiple sclerosis (MS) is a chronic immune mediated disease of the central nervous system, affecting approximately 1 in 1000 individuals. This disease is thought to be propagated by autoreactive inflammatory T cells including Th1 and Th17 cells. Experimental autoimmune encephalomyelitis (EAE), a MS disease model in rodents, shares clinical and pathological features with MS. Although some treatments are effective in MS, a large patient population suffers intolerable side effects, relapse, or fails to respond altogether. Therefore, the identification of novel therapeutic targets is warranted.

**Results**

The immunoproteasome is a cytokine-induced variant of the 20S proteasome bearing the unique subunits LMP2 (β1i), MECL-1 (β2i), and LMP7 (β5i). Recently, a novel role of the immunoproteasome in promoting the differentiation of pro-inflammatory T helper cells in chronic inflammation has been identified. In this study we have investigated the impact of the LMP7-selective inhibitor ONX 0914 on two mouse models for MS. ONX 0914 strongly attenuated disease progression in diseased animals following immunization with autoantigen or transfer of autoreactive T cells. Isolation of lymphocytes from the brain or spinal cord at the peak of the disease revealed a strong reduction in cellular infiltration and of cytokine-producing CD4^+^ cells in ONX 0914 treated mice. Recent work has demonstrated a crucial role for GM-CSF in MS. ONX 0914 reduced GM-CSF production of activated mouse T cells and in human peripheral blood mononuclear cell (PBMC) stimulated through the T cell receptor. An analysis of draining lymph nodes after induction of EAE revealed that the differentiation to Th17 or Th1 cells was strongly impaired in ONX 0914 treated mice. Consecutive episodes of remission and relapse are hallmarks of MS in the majority of patients. ONX 0914 strongly attenuated clinical symptoms in a relapsing-remitting EAE model and prevented a second exacerbation when treatment started in the recovery phase after the first wave of symptoms.

**Impact**

Our results strongly implicate the immunoproteasome in the development of EAE and suggest that immunoproteasome inhibitors are promising drug targets for the treatment of MS. Our data argue that especially autoimmune diseases which rely on a strong Th1 and Th17 driven pro-inflammatory response should be amenable to therapy with LMP7 inhibitors. These advances in characterizing LMP7 as a drug target for the therapy of autoimmune diseases will promote the further development of LMP7 inhibitors and the clinical testing of ONX 0914 for the suppression of autoimmunity.

### Histological analysis

Spinal cords were fixed in 4% formalin in phosphate buffer (55 mM Na_2_HPO_4_, 12 mM NaH_2_PO_4_). The fixed tissues were dehydrated and embedded in paraffin. Seven-micrometer sections were stained with H&E and scored in a blinded manner. The degree of inflammation on microscopic cross-sections of the spinal cord was graded semiquantitatively: 0, none; 1, minimal; 2, mild; 3, moderate; and 4, severe.

### T cell stimulation

Splenocytes of C57BL/6 (1.5 × 10^5^ per well) were incubated with ONX 0914 and stimulated with plate bound anti-CD3 (5 μg/ml, clone 17A2; eBioscience, San Diego, CA, USA) and anti-CD28 (5 μg/ml, clone 37.51; BD Biosciences, Heidelberg, Germany) antibodies for 24 h. GM-CSF in the supernatant was determined by ELISA, according to the manufacturer's protocol (eBioscience). For GM-CSF polarizing conditions, anti-IL-12 (10 μg/ml, clone C15.6; BD Biosciences) and anti-IFN-γ (10 μg/ml, clone XMG1.2; eBioscience) antibodies were added and supernatants were analyzed 96 h post stimulation.

Human PBMC′s from healthy volunteers were incubated with ONX 0914 for 2 h at 37°C. Washed cells (2.5 × 10^5^ per well) were stimulated with plate bound anti-CD3 (1 μg/ml, clone OKT3; eBioscience) and anti-CD28 (5 μg/ml, clone CD28.2; BD Biosciences) antibodies for 24 h. GM-CSF or IL-23 in the supernatant was determined by ELISA, according to the manufacturer's protocol (eBioscience).

### Isolation of mononuclear cells from CNS

Mice were sacrificed and perfused with cold PBS to minimize contamination of brain and spinal cord with blood-derived leukocytes of peripheral blood. Mononuclear cells from the brain or spinal cord were isolated by enzymatic digestion of tissues with collagenase D (0.2 mg/ml) and DNase I (0.2 mg/ml) followed by Percoll® gradient centrifugation.

### Flow cytometry

Flow cytometry was performed exactly as previously described (Basler *et al*, 2004). Abs to CD4 (clone GK1.5) and CD45 (clone 30-F11) were obtained from eBioscience and antibodies to CD4 (clone RM4-5) and CD11b (clone M1/70) were purchased from BD Biosciences. Cells were acquired with the use of the Accuri 6 flow cytometer system.

### Intracellular cytokine staining (ICS)

Analysis of T cell responses was performed as previously detailed (Basler *et al*, 2009). Briefly, lymph node cells or mononucleated cells purified from brain or spinal cord were incubated in round-bottom 96-well plates with 10 μg/ml MOG_35–55_ peptide in 100 μl IMDM 10% plus brefeldin A (10 μg/ml) for 5–6 h at 37°C. The first 2 h of stimulation were performed in the absence of brefeldin A. The staining, fixation, and permeabilization of the cells were performed exactly as detailed previously (Basler & Groettrup, 2007). Antibodies to CD4 (clone GK1.5), GM-CSF (clone MP1-22E9), and IL-17A (clone eBio17B7) were obtained from eBioscience and antibodies to IFN-γ (clone AN-18) and TNF-α (MP6-XT22) were purchased from BD Biosciences.

### Statistical analysis

The statistical significance was determined using the Student *t*-test or ANOVA. For ANOVA, we used the Bonferroni *post hoc* analysis to compare treatment groups. All statistical analyses were performed using GraphPad Prism Software (version 4.03) (GraphPad, San Diego, CA, USA). Statistical significance was achieved when *P* < 0.05. * *P* < 0.05; ** *P* < 0.01; *** *P* < 0.001.
