# Supplementary material for: Inhibition of the immunoproteasome ameliorates experimental autoimmune encephalomyelitis
Source: EMBO Mol Med. 2014 Jan 7;6(2):226–38. doi: 10.1002/emmm.201303543 (PMC3927957; doi:10.1002/emmm.201303543)
Supplement: Supplementary file 1 [file emmm0006-0226-sd1.pdf]

EMBO Molecular Medicine

Inhibition of the immunoproteasome ameliorates experimental autoimmune encephalomyelitis (EAE)

Michael Basler, Sarah Mundt, Tony Muchamuel, Carlo Moll, Jing Jiang, Marcus Groettrup and Christopher J. Kirk

Corresponding author: Michael Basler, University of Konstanz

Review timeline:

Submission date:	26 July 2012
Editorial Decision:	28 August 2012
Resubmission:	03 October 2013
Editorial Decision:	22 October 2013
Revision received:	04 November 2013
Accepted:	05 November 2013

Transaction Report:

(Note: With the exception of the correction of typographical or spelling errors that could be a source of ambiguity, letters and reports are not edited. The original formatting of letters and referee reports may not be reflected in this compilation.)

Editors: Anneke Funk / Céline Carret

1st Editorial Decision 28 August 2012

Thank you for the submission of your manuscript "Inhibition of the immunoproteasome strongly ameliorates experimental autoimmune encephalomyelitis (EAE)". We have now heard back from the three referees whom we asked to evaluate your manuscript. As you will see, the referees acknowledge that the overall topic of the manuscript is potentially interesting. However, they also raise significant concerns, which, I am afraid, preclude publication of the manuscript in EMBO Molecular Medicine.

As you will see, the referees appreciate the relevant topic. However, they also raise important criticisms, in terms of both the conceptual advance and conclusiveness of the results. The reviewers feel that the findings are not sufficiently well supported by the data and that extensive additional experiments would be needed to address the concerns. As such, I am afraid that the referees would not support publication of the manuscript. Thus, we feel that the level of support provided by the reviewers remains too limited and I see no choice but to return the manuscript with the message that we cannot offer to publish it at this stage.

Given the potential interest of the findings, we would, however, have no objection to consider a new manuscript on the same topic if at some time in the near future you obtained data that would considerably strengthen the study and convincingly address the reviewers' concerns. I would like to stress, however, that if you were to send a new manuscript, this would be treated as a new submission rather than a revision and would be reviewed afresh, in particular with respect to the literature and the novelty of your findings at the time of resubmission. If you decide to follow this route, please nevertheless upload a letter of response to the reviewers' comments.

I am sorry to have to disappoint you at this stage but hope that this negative decision does not prevent you from considering EMBO Medicine for the publication of future studies and that the reviewers' comments are helpful in your continued work in this area.

***** Reviewer's comments *****

Referee #1:

In their study, Basler et al show that ONX 0914, a selective inhibitor of Lmp7, which is part of the immunoproteasome, inhibits the development of disease in a mouse model of multiple sclerosis, termed EAE. In addition, treatment with ONX 0914 after disease induction reduces symptoms of disease and this drug is also able to prevent relapses in a relapsing-remitting model of EAE.
This study is very interesting as it shows that proteasome inhibitors can effectively treat autoimmunity, and not only cancers.
Apart from a few comments I list below, I do have a main concern, and I guess this concerns also the authors since they were very open about it. The question is basically, how come an Lmp7-specific inhibitor is so affective, but Lmp7 deficient mice are completely susceptible to the disease? Even more, the authors could show that treatment that was affective in wild type mice was useless in Lmp7 deficient mice, indicating clearly that indeed ONX 0914 is specifically interacting with this subunit.
Be that as it may, it might be very difficult to explain, but the authors should make a bigger effort here. In addition, they may also use this characteristic of the system, namely that ONX 0914 is not effective in Lmp7 deficient mice, to better understand which cells are important in the treatment. The authors suggest that the drug functions on T cells directly, but they do not clearly show that. I suggest therefore performing at least one of the next experiments:
1. Generate MOG-specific T cells in wild type or Lmp7 KO mice, and transfer them to wild type of Lmp7 KO mice and then treat these with the drug. As the drug will only function on wild type cells, it should clarify which cells respond to it.
2. Making bone marrow chimera mice. Here, the authors should take Lmp7 KO or wild type mice and reconstitute the with wild type of Lmp7 KO bone marrow, so that the immune system of the chimera is different form the rest of the mouse, and then induce disease and treat the mice. This should again clarify which cells are responding to the drug, are these immune system cells or tissue cells.
In addition, as I wrote above, I have minor points:
1. In figure 3, can the authors explain why they see hardly any microglia cells in the treated mice and the naive mice? Normally one find normal number of these cells, which are CD11b+CD45low (in contrast to macrophages that are CD45high).
2. Why do the authors analyze RNA of cytokines in the whole CNS tissue, and when they do that, how come they find that there is quite substantial levels of IL-17 RNA, even in conditions where there are hardly any immune cells which should be the main source of this cytokine?
3. Can the authors provide at least one assay where T cells treated with the drug respond to normally? For example, they show that these cells do not differentiate well to Th1 and Th17. What about regulatory T cells and what about proliferation?
4. The authors refer too much in their discussion to figures of the paper. The discussion should not contain so much reference to figures and should be more general.

Referee #2 (Comments on Novelty/Model System):

The quality of the work is high. The authors use two EAE models, both appropriate for MS, and explore the effect of the immunoproteasome inhibitor in several contexts. The novelty is high for EAE/MS, but only medium overall, as the efficacy of the inhibitor has already been demonstrated in several animals models of inflammatory disease. Thus the impact for the nonspecialist is slightly reduced. It is still unclear what might be the link between inhibiting the immunoproteasome and reducing an inflammatory disease.

Referee #2 (Other Remarks):

The authors show that inhibition of the immunoproteasome, specifically the LMP7/b5i subunit, with a chemical inhibitor prevents or ameliorates EAE in mice. The overall incidence of disease is reduced, as are clinical severity scores. The inhibitor, ONX 0914, works in two different mouse models, one using immunization with MOG, and the other PLP. Disease inhibition is accompanied by a reduction in CD4+ T cells and their cytokines in treated mice, and reduced differentiation to both Th1 and Th17 lineages. The authors conclude that the immunoproteasome is involved in the etiology and symptomology of EAE, and that immunoproteasome inhibitors may be promising drugs for the treatment of MS.

This study adds to the growing list of immune-mediated inflammatory diseases that can be ameliorated with the immunoproteasome-specific inhibitor, ONX 0914. Previous studies have shown similar inhibition of disease in animal models of arthritis, diabetes, inflammatory bowel disease and lupus.

The results are clearly presented and provide convincing evidence of disease inhibition.

The limitations of this work are that the molecular mechanism(s) remains undefined. In addition, I do not agree with the authors' claim that this work demonstrates that the immunoproteasome is involved in the etiology of EAE. In fact, the development of EAE in LMP7-deficient mice provides direct evidence against this. Although the authors discuss the fact that LMP7-deficiency affects the incorporation of other inducible subunits (LMP2 and MECL-1) into immunoproteasomes, implying there may be other abnormalities, I fail to see how this would effectively replace the functional loss of LMP7. I would argue that the immunoproteasome is involved in pathogenesis.

In Figure 1 the axis label is missing.

Referee #3:

This is an important study demonstrating activity of LMP7 inhibitor ONX0914 in animal model of multiple sclerosis potentially paving the way for clinical trials in human MS patients. However, the paper is not that novel. First-generation proteasome inhibitor bortezomib is active in animal models of MS (Fissolo et al, Eur J immunol 2008; 38: 2401), a paper which is not quoted by authors. ONX0914 activity in multiple animal models of autoimmune diseases is well-documented. Give this, ONX0914 activity in MS models is not that surprising. Therefore, I feel that this well-executed study would be more appropriate for a specialized journal.

Resubmission 03 October 2013

Referee #1:

In their study, Basler et al show that ONX 0914, a selective inhibitor of Lmp7, which is part of the immunoproteasome, inhibits the development of disease in a mouse model of multiple sclerosis, termed EAE. In addition, treatment with ONX 0914 after disease induction reduces symptoms of disease and this drug is also able to prevent relapses in a relapsing-remitting model of EAE.

This study is very interesting as it shows that proteasome inhibitors can effectively treat autoimmunity, and not only cancers.

Apart from a few comments I list below, I do have a main concern, and I guess this concerns also the authors since they were very open about it. The question is basically, how come an Lmp7-specific inhibitor is so affective, but Lmp7 deficient mice are completely susceptible to the disease? Even more, the authors could show that treatment that was affective in wild type mice was useless in

Lmp7 deficient mice, indicating clearly that indeed ONX 0914 is specifically interacting with this subunit.

Reply: To address the question why an LMP7-specific inhibitor is so effective, but LMP7-deficient mice are completely susceptible to the disease, the following experiment was performed: EAE was induced in LMP7-deficient mice and the mice were treated with the ß5c (constitutive counterpart of LMP7)-selective inhibitor PR-825 (described in Muchamuel et al., Nat Med., 2009;15(7):781-7). LMP7-deficient mice treated with PR-825 were, in contrast to vehicle treated mice, almost completely protected from EAE (Fig. 2D revised manuscript). In contrast, C57BL/6 wild type mice show no effect when treated with PR-825 (see figure 2C in the manuscript). Hence, we can experimentally explain the discrepancy in phenotype between drug-treated mice and immunoproteasome knockout mice. Namely, the chymotrypsin-like activity of the proteasome is responsible for the observed beneficial effects of the LMP7-selective inhibitor ONX 0914 on EAE. It appears that the cells responsible for the induction of EAE predominantly contain immunoproteasomes. Therefore, the chymotrypsin-like activity in wild type cells (contain LMP7) can be largely blocked with ONX 0914, whereas in LMP7-deficient mice (incorporate ß5c instead of LMP7 into immunoproteasomes), this activity can be sufficiently inhibited by the ß5c-specific inhibitor PR-825. We included this figure into the manuscript (Fig. 2D). This is a very important finding because it provides the rationale why LMP7 inhibitors which, in contrast to broad spectrum proteasome inhibitors like bortezomib, can be applied far below the maximally tolerated dose whereas bortezomib has to be applied in the clinics at the maximally tolerated dose entailing the known severe side effects.

Be that as it may, it might be very difficult to explain, but the authors should make a bigger effort here. In addition, they may also use this characteristic of the system, namely that ONX 0914 is not effective in Lmp7 deficient mice, to better understand which cells are important in the treatment. The authors suggest that the drug functions on T cells directly, but they do not clearly show that. I suggest therefore performing at least one of the next experiments:

1. Generate MOG-specific T cells in wild type or Lmp7 KO mice, and transfer them to wild type of Lmp7 KO mice and then treat these with the drug. As the drug will only function on wild type cells, it should clarify which cells respond to it.

Reply: We generated MOG-specific $CD4^+$ T cells from wild type and LMP7 KO mice. The cells were transferred into WT mice (d0) and treated on d0 and d2 with pertussis toxin. Whereas wild type cells induced EAE in host mice, no signs of EAE were detectable in mice receiving cells from LMP7 KO mice. The experiment was repeated several times with the same outcome.

It was previously reported that adoptively transferred LMP7 KO cells do not expand in wild type mice upon viral infection (Chen et al., J Exp Med., 2001;193(11):1319-26, Moebius et al., Eur J Immunol., 2010;40(12):3439-49). In our opinion, similar to the viral infection model, adoptively transferred LMP7 KO cells do not expand in wild type host mice in EAE. Hence, the requested experiment is technically not feasible. Nevertheless, as suggested by the referee we performed the next experiment (see below, next point).

2. Making bone marrow chimera mice. Here, the authors should take Lmp7 KO or wild type mice and reconstitute the with wild type of Lmp7 KO bone marrow, so that the immune system of the chimera is different form the rest of the mouse, and then induce disease and treat the mice. This should again clarify which cells are responding to the drug, are these immune system cells or tissue cells.

Reply: In order to address this issue, wild type mice were irradiated and reconstituted with either wild type or LMP7-deficient bone marrow. Mice were immunized with MOG_{35-55} and ONX 0914 or vehicle was administered three times a week at an LMP7-selective concentration of 10 mg/kg (see Figure 2E revised manuscript). ONX 0914 treatment only reduced the clinical score in mice reconstituted with wild type bone marrow but not in wild type mice reconstituted with LMP7-

deficient bone marrow. Hence, we conclude that cells derived from the hematopoietic system are responsible for the amelioration of EAE symptoms in ONX 0914 treated mice. We included this important result into the manuscript (Fig. 2E revised manuscript).

In addition, as I wrote above, I have minor points:

1. In figure 3, can the authors explain why they see hardly any microglia cells in the treated mice and the naive mice? Normally one find normal number of these cells, which are CD11b+CD45low (in contrast to macrophages that are CD45high).

Reply: In figure 3A (flow cytometry dot plot) the not evaluated $CD11b^{+}CD45^{low}$ population can readily be detected in the brain of treated and naïve mice below the gated $CD11b^{hi}CD45^{hi}$ population. In the spinal cord this population is more difficult to observe, although clearly detectable. Since the total number of mononuclear cells recovered from treated and naïve CNS (after percoll gradient centrifugation) is much lower compared to diseased mice, only low numbers of $CD11b^{+}CD45^{low}$ cells are visible.

2. Why do the authors analyse RNA of cytokines in the whole CNS tissue, and when they do that, how come they find that there is quite substantial levels of IL-17 RNA, even in conditions where there are hardly any immune cells which should be the main source of this cytokine?

Reply: The mRNA levels of proinflammatory cytokines like IL17A or TNF-a are reliable indicators of inflammation in the CNS (Fig. 3C), which are also used in other EAE studies. It appears that the few $CD4^{+}$ cells we find in the CNS (e.g. after ONX 0914 treatment) are enough to produce reproducibly detectable quantities of IL-17A and TNF-a mRNAs.

3. Can the authors provide at least one assay where T cells treated with the drug respond to normally? For example, they show that these cells do not differentiate well to Th1 and Th17. What about regulatory T cells and what about proliferation?

Reply: We recently published that ONX 0914 treatment increases differentiation of naïve CD4+ to regulatory T cells while the differentiation to Th1 and Th17 cells was inhibited (see Figs. 4A and 5E in Kalim et al., J Immunol., 2012;189(8):4182-93). Additionally, we could demonstrate that ONX 0914 has no influence on Th2 differentiation (see Fig. 3D-F in Kalim et al.).

4. The authors refer too much in their discussion to figures of the paper. The discussion should not contain so much reference to figures and should be more general.

Reply: We modified the discussion according to the recommendation of our referee.

Referee #2 (Comments on Novelty/Model System):

The quality of the work is high. The authors use two EAE models, both appropriate for MS, and explore the effect of the immunoproteasome inhibitor in several contexts. The novelty is high for EAE/MS, but only medium overall, as the efficacy of the inhibitor has already been demonstrated in several animal models of inflammatory disease. Thus the impact for the nonspecialist is slightly reduced. It is still unclear what might be the link between inhibiting the immunoproteasome and reducing an inflammatory disease.

Reply: The “link between inhibiting the immunoproteasome and reducing an inflammatory disease” is the following: Immunoproteasome inhibition reduces Th1 and Th17 differentiation and proinflammatory cytokine production (Kalim et al., J Immunol., 2012;189(8):4182-93; Muchamuel et al., Nat Med., 2009;15(7):781-7; and this manuscript). Thereby, the inflammatory response is reduced.

Referee #2 (Other Remarks):

The authors show that inhibition of the immunoproteasome, specifically the LMP7/b5i subunit, with a chemical inhibitor prevents or ameliorates EAE in mice. The overall incidence of disease is reduced, as are clinical severity scores. The inhibitor, ONX 0914, works in two different mouse models, one using immunization with MOG, and the other PLP. Disease inhibition is accompanied by a reduction in CD4+ T cells and their cytokines in treated mice, and reduced differentiation to both Th1 and Th17 lineages. The authors conclude that the immunoproteasome is involved in the etiology and symptomology of EAE, and that immunoproteasome inhibitors may be promising drugs for the treatment of MS.

This study adds to the growing list of immune-mediated inflammatory diseases that can be ameliorated with the immunoproteasome-specific inhibitor, ONX 0914. Previous studies have shown similar inhibition of disease in animal models of arthritis, diabetes, inflammatory bowel disease and lupus.

The results are clearly presented and provide convincing evidence of disease inhibition.

The limitations of this work are that the molecular mechanism(s) remains undefined.

Reply: ONX 0914 prevented the differentiation of naïve $CD4^+$ cells to Th1 and Th17 in MOG_{35-55} immunized mice (Fig. 3D, 5E). Last year we have reported that the phosphorylation of STAT1 and STAT3 during Th1 and Th17 differentiation from naïve T cells in vitro is suppressed in the presence of ONX 0914 (Kalim et al., J Immunol., 2012;189(8):4182-93). It is well established that these T cell populations are responsible for the development of EAE. Additionally, we demonstrated that ONX 0914 reduced GM-CSF expression of mouse and human T cells (Fig. 4). It has been shown by others that T cell-derived GM-CSF sustains neuroinflammation via myeloid cells that infiltrate the CNS. Additionally, with the newly inserted Figure 2D we provide a new mechanism how proteasome inhibition influences EAE (see above).

In addition, I do not agree with the authors' claim that this work demonstrates that the immunoproteasome is involved in the etiology of EAE. In fact, the development of EAE in LMP7-deficient mice provides direct evidence against this. Although the authors discuss the fact that LMP7-deficiency affects the incorporation of other inducible subunits (LMP2 and MECL-1) into immunoproteasomes, implying there may be other abnormalities, I fail to see how this would effectively replace the functional loss of LMP7. I would argue that the immunoproteasome is involved in pathogenesis.

Reply: As we point out in our reply to referee #1 above, the contribution of LMP7 to the chymotrypsin-like activity of the proteasome in immune cells with high LMP7 content is required for the development of EAE (see Fig. 2D). We removed the word etiology from the abstract and the discussion.

In Figure 1 the axis label is missing.

Reply: We inserted the axis label.

Referee #3:

This is an important study demonstrating activity of LMP7 inhibitor ONX0914 in animal model of multiple sclerosis potentially paving the way for clinical trials in human MS patients. However, the paper is not that novel. First-generation proteasome inhibitor bortezomib is active in animal models of MS (Fissolo et al, Eur J immunol 2008; 38: 2401), a paper which is not quoted by authors.

Reply: Broad-spectrum proteasome inhibitors like bortezomib are probably of no clinical relevance in the treatment of chronic autoimmune diseases due to their strong and undesired side effects. In contrast, ONX 0914 is selectively targeting the chymotrypsin-like activity in cells originating from the hematopoietic system (like T cells, dendritic cells, or myeloid cells) expressing high levels of immunoproteasomes and these cells are centrally involved in the induction of EAE. Hence, ONX 0914 is selectively targeting the chymotrypsin-like activity at the site of inflammation while the chymotrypsin-like activity required for housekeeping functions of the proteasome in uninflamed tissues is barely affected. This explains the lacking side effects of ONX 0914 in mice compared to broad-spectrum proteasome inhibitors.

The application of bortezomib to ameliorate EAE in the study by Fissolo et al. can not address which of the subunits of the constitutive proteasome and the immunoproteasome are required for disease development. The demonstration that the application of ONX 0914 in an LMP7 selective dose ameliorates the course and severity of EAE identifies the subunit LMP7 as a pivotal target. This is a major advance because it sets the stage for clinical testing of LMP7 selective inhibitors as potential therapeutics in multiple sclerosis patients as ONX 0914 has much less side effects than bortezomib at least in mice. In the revised version of our manuscript we discuss these issues and cite the paper by Fissolo et al.

ONX0914 activity in multiple animal models of autoimmune diseases is well-documented. Give this, ONX0914 activity in MS models is not that surprising.

Reply: While some pathogenic principles are probably shared among different pre-clinical models of autoimmune diseases others are not. This is evidenced by the diverging effectivity of immunsuppressive drugs against different kinds of autoimmune diseases. It is therefore not possible to extrapolate the efficacy of LMP7 inhibition from e.g. experimental colitis or lupus – like disease to EAE. In fact, the data on DSS induced colitis (Basler et al. (2010) J. Immunol. 185: 634) show that LMP7 inhibition and LMP7 knock out both protect from the disease. As this is not the case for EAE, different pathogenetic mechanisms are very likely involved in EAE and DSS colitis as evidenced in the revised figure 2.

Therefore, I feel that this well-executed study would be more appropriate for a specialized journal.

Reply: This manuscript describes for the first time LMP7 as a pivotal target in preventing EAE. On conferences where we presented our data we have experienced a very strong interest of scientists from pharmaceutical industry, neuroscientists, and neurologists in LMP7 inhibition as a new treatment option for multiple sclerosis. Our study on LMP7 inhibition in experimental arthritis (Muchamuel et al. (2009) Nature medicine 15:781) has been cited 100 times supporting the interest in this new approach.

2nd Editorial Decision 22 October 2013

Thank you for the submission of your revised manuscript to EMBO Molecular Medicine. We have now received the enclosed reports from the referees that were asked to re-assess it. As you will see the reviewers are supportive and I am pleased to inform you that we will be able to accept your

manuscript pending the following final amendments:

1) we would encourage you to comply with referee 2's suggestion and modify the title accordingly

2) during figures inspection we noticed that figure 3 is very busy, consequently the internal labelling in some panels cannot be read. Please reorganise the figures and make sure that the new organisation is called properly within the main text and that all labels are clearly visible and readable even if the final figure should be reduced.

Please submit your revised manuscript within two weeks. I look forward to seeing a revised form of your manuscript.

***** Reviewer's comments *****

Referee #1 (Comments on Novelty/Model System):

The additional data provided in the revised manuscript showing that PR-825 protects LMP7-/-, but not wild type mice (and the opposite result using ONX 0914), add novelty, inferred mechanism, and impact to the paper. They further the pre-clinical advancement of these compounds, and provide a rationale for their potential use in other inflammatory diseases.

Referee #1 (Remarks):

The authors have carefully considered the reviewers' concerns and responded with additional experiments and modifications that substantially improve the clarity of the science and the manuscript.

Referee #2 (Comments on Novelty/Model System):

The article is very interesting and is novel in that that it propose a new therapy for CNS inflammation diseases

Referee #2 (Remarks):

The authors have answered all my concerns. If I would need still to change one thing, it will be in the title. I really do not like the use of the word "strongly" and would remove it or replace by "significantly".

1st Revision - authors' response 04 November 2013

Please find below how we have dealt with the final amendments requested:

- 1) We changed the title to ´Inhibition of the immunoproteasome ameliorates experimental autoimmune encephalomyelitis (EAE).
- 2) Figure 3 was split into two Figures (new Figure 3 and 4).

Below you find how we have dealt with the reviewer's comments:

Referee #2 (Remarks):

The authors have answered all my concerns. If I would need still to change one thing, it will be in the title. I really do not like the use of the word "strongly" and would remove it or replace by "significantly".

We changed the title to ´Inhibition of the immunoproteasome ameliorates experimental autoimmune encephalomyelitis (EAE).
