# Supplementary material for: Inhibition of the immunoproteasome ameliorates experimental autoimmune encephalomyelitis
Source: EMBO Mol Med. 2014 Jan 7;6(2):226–38. doi: 10.1002/emmm.201303543 (PMC3927957; doi:10.1002/emmm.201303543)
Supplement: Supplementary file 2 [file emmm0006-0226-sd2.pdf]

Supplemental Information

Inhibition of the immunoproteasome ameliorates experimental autoimmune encephalomyelitis (EAE)

Michael Basler, Sarah Mundt, Tony Muchamuel, Carlo Moll, Jing Jiang, Marcus Groettrup & Christopher J. Kirk

Table of content

Supplemental Figures and Legends..1
Figure S1: Th17 status of $PLP_{139\text{-}151}$ peptide restimulated $CD4^+$ T cells..................................1

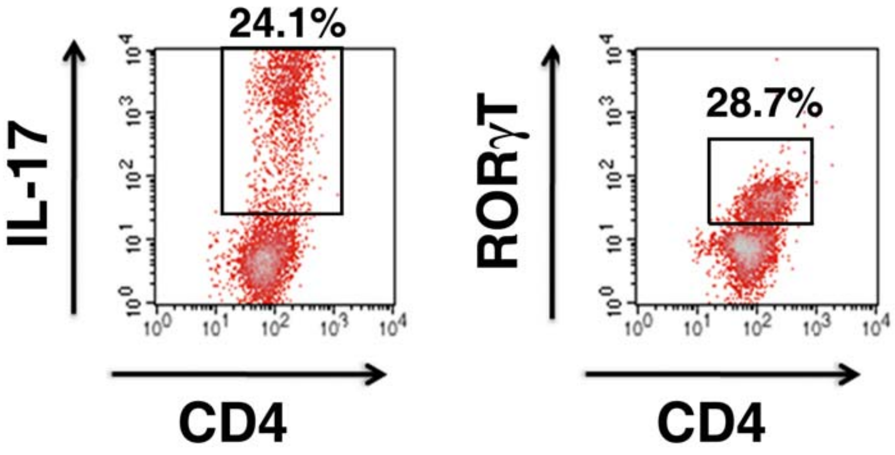

Figure S1: Th17 status of $PLP_{139\text{-}151}$ peptide restimulated $CD4^+$ T cells. DLN were harvested 8-10 days post PLP $_{139–151}$/CFA immunization and cells were cultured for four days in the presence of 20 µg/ml PLP and 5 ng/ml of rIL- 23. IL-17 production and RORγT expression in $CD4^+$ T cells was analyzed by intracellular staining.
